# Feasibility and outcomes of bronchial artery embolization in patients with non-massive hemoptysis

**DOI:** 10.1186/s12931-021-01820-x

**Published:** 2021-08-06

**Authors:** Jung Han Hwang, Jeong Ho Kim, Suyoung Park, Ki Hyun Lee, So Hyun Park

**Affiliations:** Department of Radiology, Gil Medical Center, Gachon University College of Medicine, 21, Namdong-daero 774beon-gil, Namdong-gu, Incheon, 21565 Republic of Korea

**Keywords:** Bronchial artery, Embolization, Hemoptysis, Contrast-enhanced CT, Bronchoscopy

## Abstract

**Purpose:**

To evaluate the safety, efficacy, and long-term outcome of bronchial artery embolization (BAE) in the treatment of non-massive hemoptysis and the prognostic factors associated with recurrent bleeding.

**Materials and methods:**

From March 2005 to September 2014, BAE was performed in 233 patients with non-massive hemoptysis. All patients had a history of persistent or recurrent hemoptysis despite conservative medical treatment. We assessed the technical and clinical success, recurrence, prognostic factors related to recurrent bleeding, recurrence-free survival rate, additional treatment, and major complications in all the patients.

**Results:**

Technical success was achieved in 224 patients (96.1%), and clinical success was obtained in 219 (94.0%) of the 233 patients. In addition, 64 patients (27.5%) presented hemoptysis recurrence with median time of 197 days after embolization. Tuberculosis sequelae and presence of aberrant bronchial artery or non-bronchial systemic collaterals were significantly related to recurrent bleeding (*p* < 0.05). The use of Histoacryl-based embolic materials significantly reduced the recurrent bleeding rate (*p* < 0.05). Patient who had a tuberculosis sequelae showed a significantly lower recurrence-free survival rate (*p* = 0.013). Presence of aberrant bronchial artery or non-bronchial systemic collaterals showed a statistically significant correlation with recurrence-free survival rate (*p* = 0.021). No patients had major complications during follow-up.

**Conclusions:**

BAE is a safe and effective treatment to manage non-massive hemoptysis. The procedure may offer a better long-term control of recurrent hemoptysis and quality of life than conservative therapy alone.

## Background

Hemoptysis is graded in mild, moderate, and severe (massive) levels, although the grading definitions may differ across studies [1]. Massive or life-threatening hemoptysis has been defined as bleeding more than 300 mL over 24 h [2, 3]. As it corresponds to an emergency that requires prompt treatment, bronchial artery embolization (BAE) is an effective life-saving procedure for patients presenting massive hemoptysis [4–10]. On the contrary, non-massive hemoptysis can often be managed by conservative treatment of the underlying pathology which means treatment of the infection or anti-inflammatory measures [11].

Most previous studies on BAE have been focused on controlling massive hemoptysis [3]. BAE can be used as the last option after conservative management in patients with non-massive hemoptysis. In general, physicians recommend conservative treatments for non-massive hemoptysis [12]. Nevertheless, such treatments may lead to chronic recurrent hemoptysis and undermine long-term survival [12, 13]. The mortality rate is 5–21% even in non-massive hemoptysis with benign or malignant underlying causes [14, 15]. In several case studies, the early treatment of non-massive hemoptysis by BAE has improved the patients’ physiological well-being and reinforced compliance with subsequent treatments [12, 16]. To the best of our knowledge, the effectiveness of BAE for patients with non-massive hemoptysis due to varying causes has not been properly investigated to date.

In this study, we aimed to retrospectively evaluate the safety, efficacy, and long-term outcomes of BAE and to determine prognostic factors associated with recurrent bleeding for the treatment of non-massive hemoptysis in patients with a variety of underlying diseases.

## Materials and methods

### Study design and study population

A single-center retrospective study was approved by the institutional review boards of the local ethics committee. The requirement to obtain written informed consent from the patients was waived due to the retrospective nature of this study. In total, 451 consecutive hemoptysis patients treated by BAE were referred to the department of interventional radiology between March 2005 and September 2014. Patient with persistent or recurrent hemoptysis were included despite conservative medical treatment. BAE was performed under the condition of standby rather than urgency. Patients with massive hemoptysis (*n* = 216) or in no clinical follow-up more than 1 month (*n* = 2) were excluded. Thus, 233 patients with non-massive hemoptysis treated by BAE were enrolled in this study. The patients’ characteristics are listed in Table 1. The 143 male and 90 female patients had ages of 56.9 ± 14.8 years at the time of their interventions. The most common cause of hemoptysis was tuberculosis sequelae, followed by active tuberculosis, bronchiectasis without tuberculosis, pneumonia, lung cancer, and other causes. The patients presented non-massive hemoptysis with volume of 88 ± 69 mL (range, 20–300 mL) over 24 h. In 147 patients (63.1%), contrast-enhanced chest CT with bronchoscopy was performed within 3 months before BAE.

**Table 1:** Demographic data of 233 patients with non-massive hemoptysis

Characteristic	Value (*N* = 233)
Age (years)	56.9 ± 14.8
Sex (male/female)	143/90
Underlying disease	
DM/HTN	17/34
Causes of non-massive hemoptysis	
TB sequelae	99 (42.5)
Bronchiectasis	31 (13.2)
Tuberculosis destroyed lung	25 (10.9)
Aspergilloma	26 (11.1)
Fibrotic scar change	17 (7.3)
Active TB	31 (13.2)
Mycobacterium tuberculosis	28 (11.9)
Multidrug-resistant tuberculosis	3 (1.3)
Nontuberculous mycobacteria	8 (3.3)
Bronchiectasis without TB	35 (15.2)
Pneumonia	27 (11.6)
Lung cancer	11 (4.6)
Others	22 (9.6)
Amount of hemoptysis (mL)	
< 50	92 (39.5)
50–100	81 (34.7)
101–200	57 (24.5)
201–300	3 (1.3)
Pre-procedural evaluation^a^	
Contrast-enhanced CT + Bronchoscopy	147 (63.1%)
Contrast-enhanced CT	72 (31.0%)
Bronchoscopy	3 (1.2%)
None	11 (4.7%)
In-hospital mortality	
Yes^b^	5 (2.1)
No	228 (97.9)

Values in parentheses represent percentages. ^a^Contrast-enhanced chest CT and bronchoscopy were performed within 3 months before BAE. ^b^Five patients died following complications of underlying diseases (heart failure, 3 patients; multiorgan failure, 2 patients). CT, computed tomography, DM, diabetes mellitus, HTN, hypertension, TB, tuberculosis

### Angiography and embolization

Two experienced interventional radiologists performed the BAE procedures. A standard technique that had been previously reported was used for the procedure [1]. If contrast-enhanced CT scans were available for a patient, the radiologist reviewed the vessel anatomy (i.e., bronchial artery origin, non-bronchial systemic collaterals, and aberrant bronchial artery) and determined the target vessel before the procedure. The 1 mm CT raw data was reviewed using a three-dimensional software for evaluation of the bleeding arteries. After subcutaneous administration of 2% lidocaine, the femoral artery was punctured with a 21G micropuncture needle (Cook Medical) under ultrasonographic guidance, and a fluoroscopic image was obtained after insertion of a 0.018-inch guide wire (Silhouette; Cook Medical). After dilatation of the puncture site with a microdilator (Silhouette; Cook Medical) and insertion of a 0.035-inch hydrophilic guide wire (Terumo), a 5 French Cobra catheter (C2; Cook Medical) was the most commonly used to select the bronchial artery and non-bronchial systemic collateral branches from the internal mammary, subclavian, and axillary arteries. Various types of catheters were used due to the diversity of blood vessels. The pathologic artery was concerned upon identification of tortuous hypertrophy, regions of hypervascularity, systemic-to-pulmonary shunting, or extravasation of the contrast agent [17]. BAE was then performed as follows. If the pathologic artery was identified, a 1.7–2.0-French microcatheter (Progreat; Terumo) was introduced coaxially into the 5-French catheter and advanced through the pathologic arteries at the most possible distal point to avoid reflux of embolic materials into the artery of Adamkiewicz or the aorta. After the microcatheter was inserted in the pathologic artery, embolic materials were injected. The agent for embolization was selected according to the radiologist preference and contained gelatin sponge (Gelfoam) particles (Spongostan; Johnson & Johnson), microcoils (Hilal or Tornado; Cook Medical), polyvinyl-alcohol (PVA) particles (Bearing; Merit Medical), and/or N-butyl-2-cyanoacrylate (NBCA; B. Braun Medical).

### Definition and study endpoint

We assessed the technical success, clinical success, recurrence, additional treatments, and major complications in all the patients. The technical success was defined as the cessation and exclusion of the bleeding focus from the bronchial artery after embolization. The clinical success was defined as freedom of hemoptysis for at least 1 month after embolization [18]. Recurrence was defined as recurrent hemoptysis during the clinical follow-up period and requiring immediate additional treatment such as repeated BAE or endoscopic/surgical treatment. The electronic medical records of each patient were analyzed to collect data on relevant aspects including patient demographic characteristics, details of procedure (angiographic findings, embolized arteries and embolic materials on initial BAE), prognostic factors related to recurrent bleeding (cause of bleeding, embolic materials, and embolized arteries), recurrence-free survival rate, additional treatment, and procedure-related major complications (e.g., stroke, systemic or cardiac complications due to embolic materials).

### Statistical analysis

A chi-squared test was applied to determine the significant factors related to recurrent bleeding after embolization. The recurrence-free survival rates depending on each variable were estimated using the Kaplan-Meier method and compared using the log-rank test. All the statistical analyses were performed using a statistical software (MedCalc version 15.8; MedCalc Software).

## Results

**Fig. 1:**
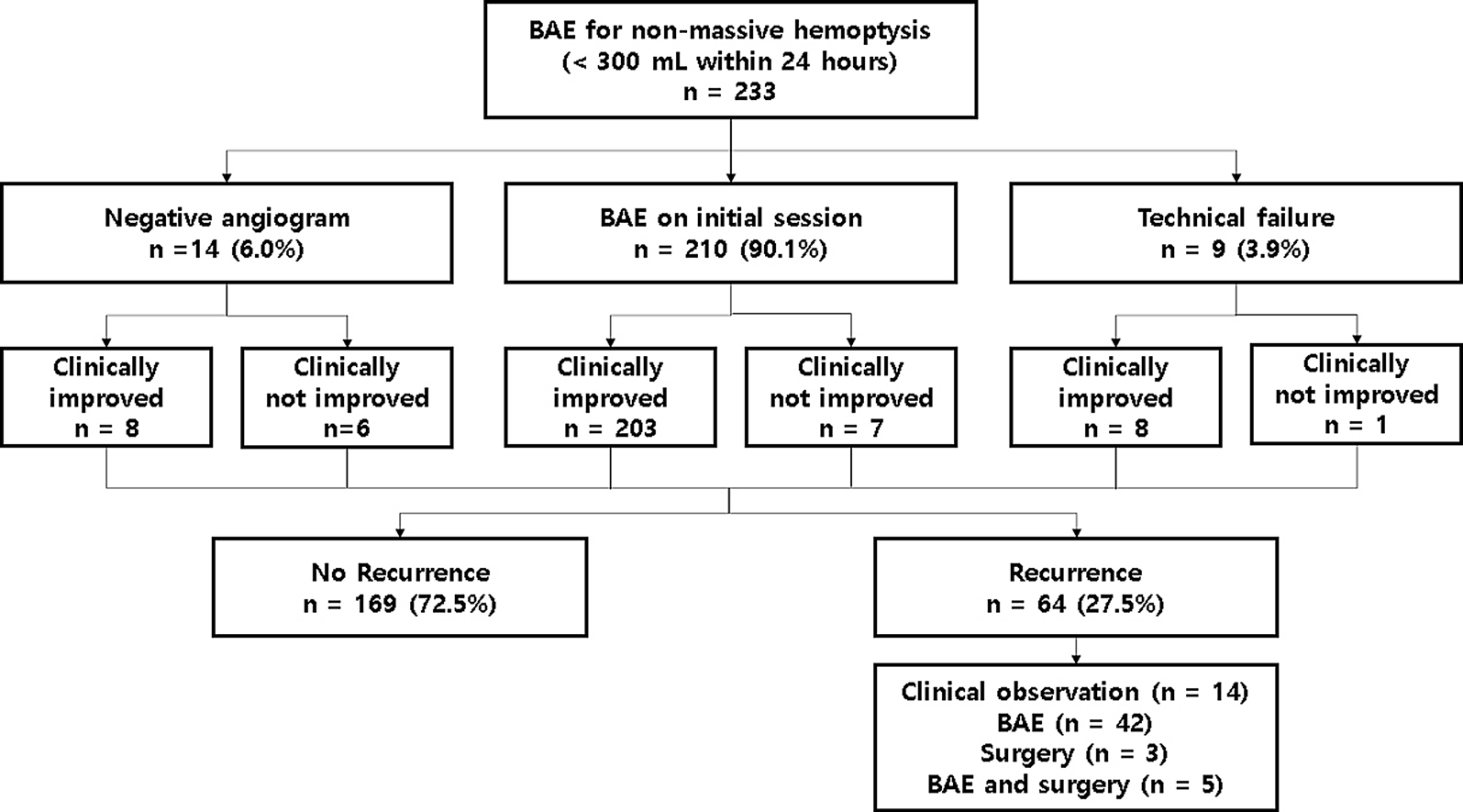
Diagram showing clinical flow of 233 patients referred for BAE

**Fig. 2:**
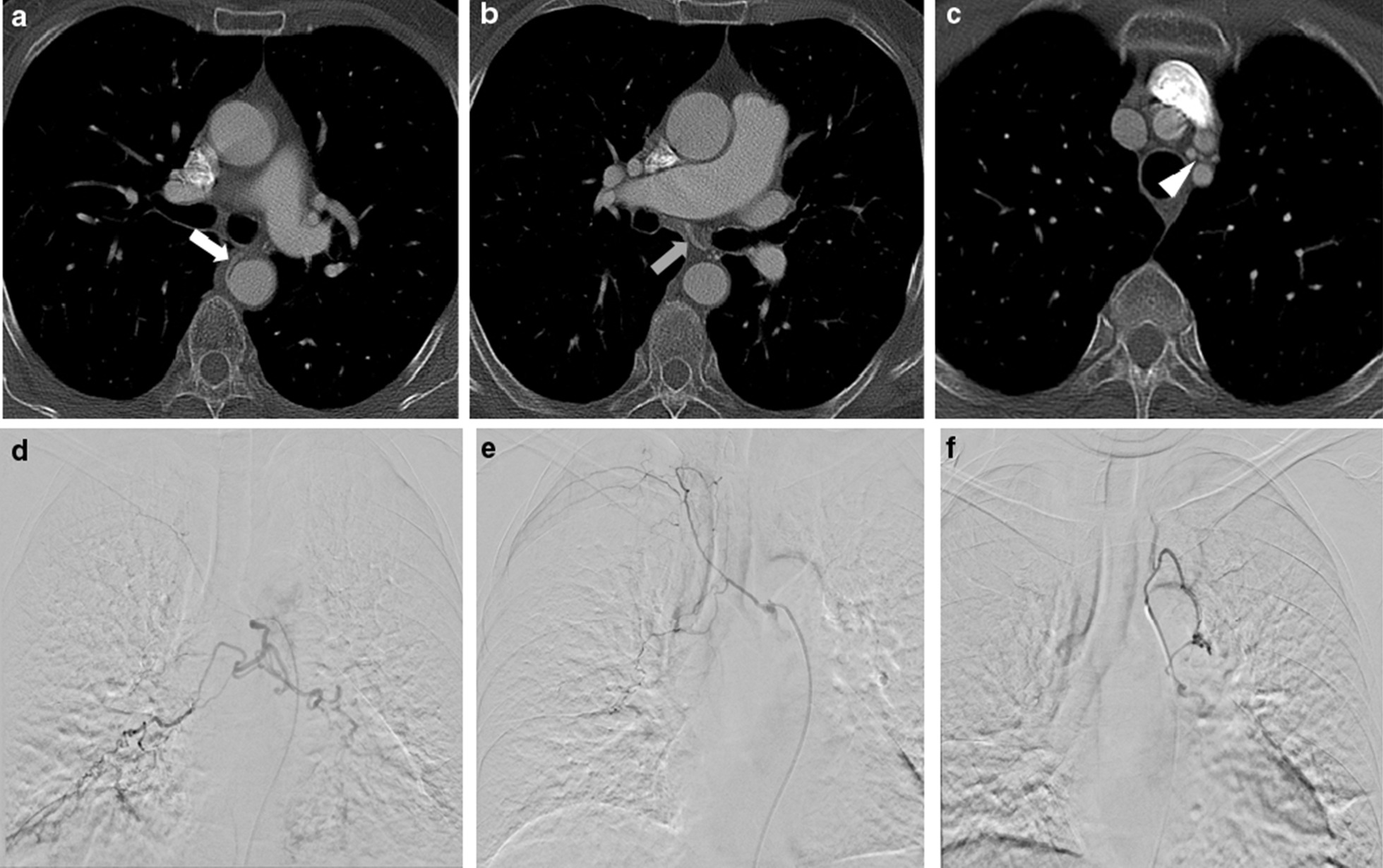
A 65-year-old male patient with known tuberculosis and prior right-upper lobe lung wedge resection now presenting with aspergillosis and hemoptysis. Preprocedural contrast-enhanced chest CT scans show a hypertrophic change of (**A**) left bronchial artery (white arrow), **B** right bronchial artery (gray arrow), and **C** aberrant left bronchial artery form left vertebral artery (arrowhead). Selective bronchial angiograms show hypertrophic change in (**D**) left bronchial artery, **E** right bronchial artery, and **F** aberrant left bronchial artery form left vertebral artery with hyperemic strains. The pre-procedural CT is consistent with the angiography findings. All hypertrophic arteries were embolized with the use of PVA and NBCA. Postembolization angiography shows no residual bleeding foci. The patient was uneventful during the follow-up period (2365 days)

The characteristics and outcomes of BAE are listed in Table 2 and Fig. 1. From the 233 patients, 14 patients did not undergo BAE due to negative identification from initial angiography, and the remaining 219 patients underwent BAE via bronchial arteries, aberrant bronchial arteries, or non-bronchial systemic collaterals. The most common origin of aberrant bronchial arteries is the aortic arch (*n* = 10). Other origins have included the subclavian artery (*n* = 7), thyrocervical trunk (*n* = 4), brachiocephalic artery (*n* = 3), and vertebral artery (*n* = 2). The non-bronchial systemic collaterals (*n* = 25) included intercostal (*n* = 10), internal mammary (*n* = 5), thyrocervical trunk (*n* = 5), lateral thoracic (*n* = 2), inferior phrenic (*n* = 2), and left gastric artery (*n* = 1). Positive angiographic findings included systemic-to-pulmonary shunt, tortuous hypertrophy and/or regions of hypervascularity, and few cases of extravasation of the contrast agent (Fig. 2). A variety of agents have been used alone or in combination for bronchial artery embolization according to the trend of the times and the preference of the operator. PVA was used most commonly and followed by Gelfoam and NBCA. A single type of embolic material was used in 111 patients, and combined embolic materials were used in the remaining 108 patients.

**Table 2:**
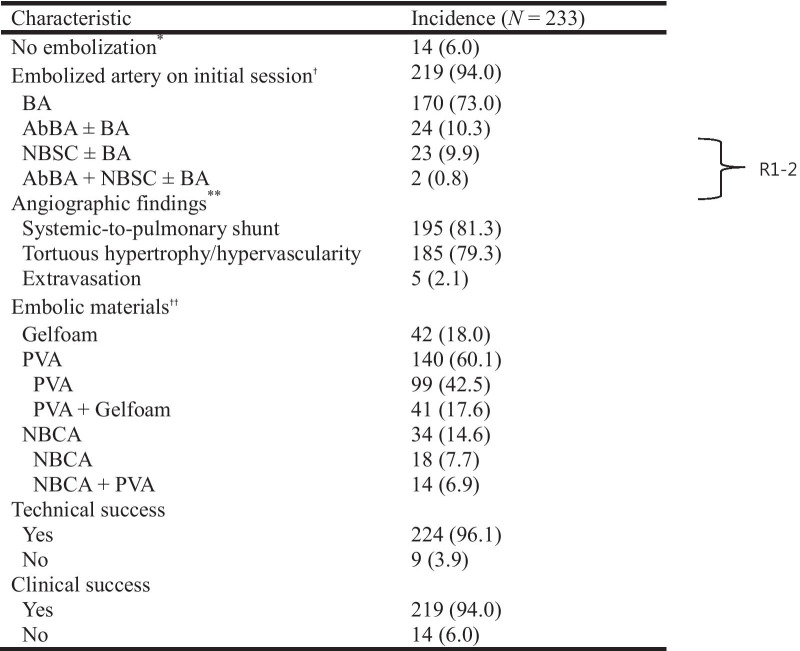
Characteristics and outcomes of BAE

Data in parentheses represent percentages. *Negative findings on initial angiography. †Diagnostic angiography to verify bleeding focus at initial session of bronchial artery embolization. **Overlapping findings. ††Microcoil used for proximal embolization of target artery in some patient. PVA, polyvinyl alcohol; NBCA, N-butyl-2-cyanoacrylate; BA, bronchial artery; AbBA, aberrant bronchial artery, NBSC, non-bronchial systemic collaterals

Technical success was obtained in 224 (96.1%) of the 233 patients. The characteristics of the 9 patients with technical failure are listed in Table 3. Technical failure was not a complete failure, but a failure of embolization of some vessel after embolization of most target vessels. The angiographic catheter could not be inserted into target arteries due to stenosis of the orifice in 8 patients. Arterial dissection occurred in 1 patient. During the follow-up period, recurrent hemoptysis appeared in 3 of these 9 patients despite the BAE completion. A second BAE was performed in 2 of the 3 patients (patients 5 and 6 in Table 3) and other one patient was clinically observed. One of the 9 patients showed no clinical improvement, whereas the other 8 patients improved eventually. One patient of lung cancer subsequently underwent surgery.

**Table 3:** Characteristics of nine patients with technical failure

Patient	Details of embolized arteries	Technical outcome	Clinical outcome
1	Right bronchial artery	Success	No clinical improvement, surgically treated^a^
	Left bronchial artery	Failure (selection failure)	
2	Right bronchial artery	Success	Improved, recurred
	Intercostal artery	Success	
	Left bronchial artery	Failure (selection failure)	
3	Both bronchial arteries	Success	Improved
	Intercostal artery	Failure (selection failure)	
4	Right bronchial artery	Success	Improved
	Left bronchial artery	Failure (selection failure)	
5	Both bronchial arteries	Success	Improved, recurred
	Right internal mammary artery	Failure (selection failure)	
6	Both bronchial arteries	Success	Improved, recurred
	Thyrocervial trunk	Success	
	Aberrant bronchial artery	Failure (selection failure)	
7	Left bronchial artery	Success	Improved
	Right bronchial artery	Failure (arterial dissection)	
8	Both bronchial arteries	Success	Improved
	Aberrant bronchial artery	Failure (selection failure)	
9	Both bronchial arteries	Success	Improved
	Left internal mammary artery	Success	
	Intercostal artery	Failure (arterial dissection)	

Technical failure was not a complete failure, but a failure of embolization of some vessel after embolization of most target vessels. ^a^The patient underwent left upper lobectomy of lung

Clinical success was obtained in 219 (94.0%) of the 233 patients. In 5 patients, control of hemoptysis could not be achieved despite the successful BAE procedures. Two patients of left-upper lobe aspergilloma subsequently underwent surgery. One patient presented persistent hemoptysis due to a failure of procedure. The other six non-responsive patients presented tumor bleeding due to lung carcinoma. These cases explain the lower clinical success compared with the technical success.

**Fig. 3:**
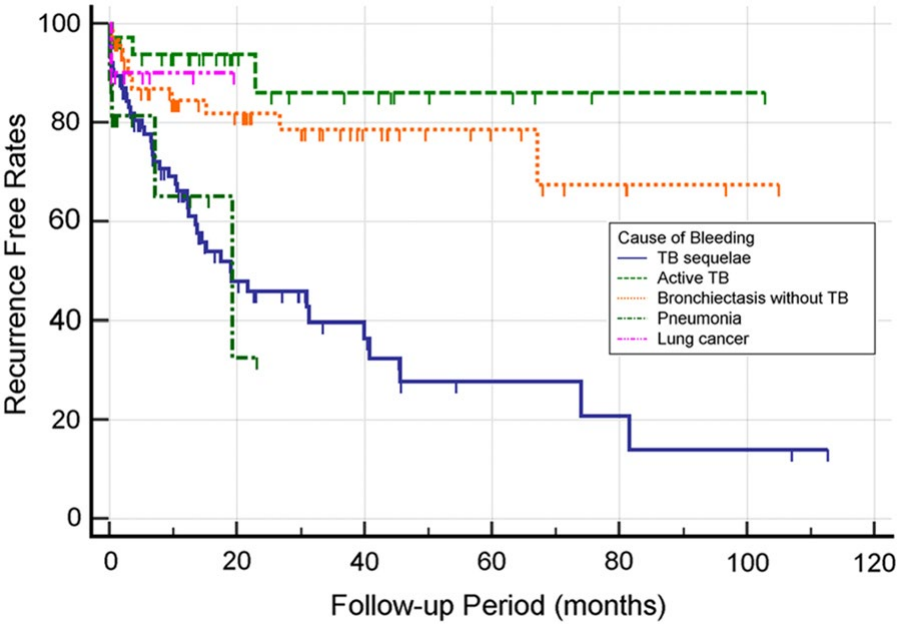
Recurrence-free survival curves correlating to cause of bleeding. The recurrence-free survival rates were highly correlated with cause of bleeding (p = 0.013), and an especially short recurrence-free time was observed in tuberculosis sequelae and pneumonia group

**Fig. 4:**
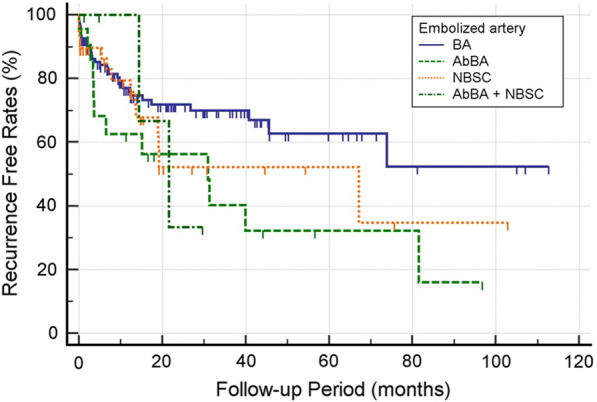
Recurrence-free survival curves correlating to embolized artery. Presence of aberrant bronchial artery or non-bronchial systemic collaterals showed a statistically significant correlation with lower recurrence-free survival rate (p = 0.021)

During or after admission, a small amount of hemoptysis (< 200 mL) recurred in 64 patients (27.5%) with median recurrence time of 197 days (range, 0–2445 days) after embolization. No massive hemoptysis occurred during the hospitalization period. From the 64 patients, 50 underwent repeated BAE (*n* = 42), surgical treatment (*n* = 3), or both (*n* = 5), and the remaining 14 patients were treated conservatively, showing subsequent disappearance of the hemoptysis. More details on recurrent bleeding are listed in Table 4. The main cause of recurrent hemoptysis was tuberculosis sequelae followed by bronchiectasis without tuberculosis, pneumonia, active tuberculosis, and lung cancer. Tuberculosis sequelae was significantly related to recurrent bleeding (*p* < 0.001). The presence of aberrant bronchial artery and non-bronchial systemic collaterals were significantly related to recurrent bleeding (*p* = 0.011 and *p* = 0.023, respectively). The use of NBCA-based embolic materials significantly reduced the recurrent bleeding rate (*p* < 0.05). The recurrence-free survival rates were highly correlated with cause of bleeding (*p* = 0.013), and an especially short recurrence-free time was observed in tuberculosis sequelae group (Fig. 3). Presence of aberrant bronchial artery or non-bronchial systemic collaterals showed a statistically significant correlation with lower recurrence-free survival rate (*p* = 0.021) (Fig. 4).

**Table 4:**
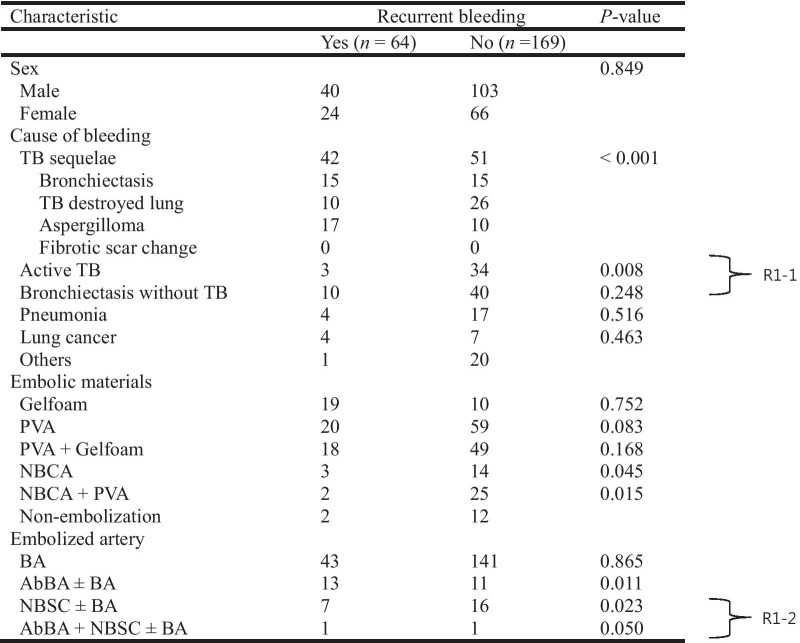
Characteristics of patients with and without recurrent bleeding after embolization

TB, tuberculosis; PVA, polyvinyl alcohol; NBCA, N-butyl-2-cyanoacrylate; BA, bronchial artery; AbBA, aberrant bronchial artery; NBSC, non-bronchial systemic collaterals

A total of 160 patients were uneventful during the follow-up period (median, 595 days; range, 32–3381 days). There were no major complications requiring immediate treatment, and only common minor complications occurred (*n* = 24). The most common symptoms were transient chest pain with mild fever (*n* = 20; 8.6%), which resolved spontaneously or upon administration of oral analgesics. Four patients presented urticaria due to hypersensitivity to the contrast media. Contrast-induced nephropathy was not observed in any patients after BAE.

## Discussion

In this study, the evaluation of 233 patients who underwent BAE to treat non-massive hemoptysis revealed that BAE is a safe and feasible treatment, a high technical and clinical success rate with low major complication rate. Before BAE, bronchoscopy and/or contrast-enhanced CT examinations were performed to identify the underlying cause and extent of pulmonary diseases, localize possible bleeding foci, and predict the culprit vessels [19]. Contrast-enhanced CT examinations were common for diagnosis (95.3%), possibly explaining the high technical success rate. These results are consistent with several studies on BAE [3–9, 13, 19–30], in which the technical success rate ranged from 81 to 100%. In our study, technical failure of BAE occurred in 9 patients whose angiographic catheter could not be inserted into the target arteries due to stenosis of the orifice. Only 1 patient did not clinically improve after BAE due to aberrant arterial supply to the lungs following recruitment of systemic blood supply and chronic inflammation. Previous studies have reported technical failure associated with incomplete embolization of existing arteries, recanalization of embolized arteries, and development of additional collateral blood supply [2, 31].

Various diseases such as pulmonary tuberculosis, bronchiectasis, and lung cancer may cause non-massive hemoptysis, with the geographic location of patients being an influential factor on the hemoptysis cause. For instance, regions of the world with an endemic burden of tuberculosis render it the main cause of hemoptysis. In fact, tuberculosis is the predominant cause of hemoptysis worldwide. In our study, the most frequent cause of non-massive bronchial bleeding was also tuberculosis, being consistent with other studies [27, 30, 32]. Furthermore, we found that tuberculosis sequelae were significantly related to recurrent bleeding. Therefore, recurrent hemoptysis tends to occur more frequently in tuberculosis patients. The recurrence rates have been postulated to have a relationship with the etiology of hemoptysis [21, 33]. In these cases, BAE should be considered as an assistant therapy to surgery or as a specific medical therapy.

Recurrent bleeding after adequate embolization remains a considerable problem, occurring in 12–57% of patients [3]. In our study, rebleeding occurred within 197 days (median) in 27.5% of the patients. A second BAE was performed in 42 patients, with 5 (11.9%) patients presenting rebleeding in the previous bleeder, whereas the others presented bleeding in other arteries, such as non-bronchial systemic collaterals. In 37 patients, additional bronchial or non-bronchial systemic collateral arteries were embolized, resulting in the successful control of hemoptysis, and 5 patients developed recanalization of the previously embolized vessels and underwent surgery. In patients with recurrent hemoptysis, tuberculosis sequelae were significantly common etiologies. The success of repeated treatment further indicates the effectiveness of BAE.

Interventional radiologists should be familiarized with prognostic factors related to recurrent bleeding. Early recurrent bleeding, within the first weeks and months after embolization, is caused by incomplete embolization of the abnormal vessels, possibly due to the extensive nature of the underlying disease or incomplete search for all abnormal vessels [5, 25, 26, 28, 30]. Advances in embolic materials have improved technical success, and the overall recurrence rates have not significantly changed since the 1970s [2, 22]. Thus, there is no consensus on the best embolic material for BAE. For the cases analyzed in this study, various embolic materials were used to perform BAE. In earlier years (2005–2008), Gelfoam-based embolic materials were predominant. In 2009–2012, PVA-based embolic materials were preferred, whereas NBCA-based embolic materials were mostly used after 2013. Gelfoam or PVA particles were most frequently used because they are easy to handle and inexpensive. Recently, the use of NBCA has been related to a significantly lower rate of recurrent bleeding, and there are more reports on the use of the liquid embolizing agent NBCA for BAE. This trend may be explained by various factors. Rapid and complete occlusion of the target vessels improves the effectiveness of embolization and reduces the procedural and fluoroscopic time, and the adjustable ratio of NBCA to iodized oil allows controlling the level of embolization within target vessels and the polymerization time [34, 35]. In addition, the use of NBCA enables both complete occlusion of the target artery and filling of the adjacent potential collateral vessels [19]. Therefore, more complete embolization is expected in hemoptysis, which usually presents multiple collateral vessels. The use of NBCA-based embolic materials significantly reduced recurrent bleeding events (*p* < 0.05) in the patients of our study.

Several limitations of this study deserve mention. First, this retrospective study only considered data from a single center, with uncertain homogeneity of data and a specific patient cohort. Nevertheless, the independent review of the database of the department of intervention radiology by three experienced radiologists improved data reliability. Furthermore, 233 consecutive patients from a single center can reduce the bias from possible confounding factors. Randomized controlled trials or prospective studies are needed to confirm the benefits of BAE in patients with non-massive hemoptysis. Second, the embolic material was selected at the discretion of the attending interventional radiologists. Therefore, the reasons for selection remain unclear, possibly biasing the results. Third, the absence of speed of hemoptysis in the study, which is important to decide the severity of disease, prevents clear definition of non-massive hemoptysis. Finally, the etiology of hemoptysis may vary among different countries, and caution is required if generalizing our results to different conditions.

## Conclusion

Our study suggests that BAE is a safe and effective treatment for non-massive hemoptysis. The procedure is well-tolerated and associated with mild complications. A good clinical outcome has been achieved in patients with non-massive hemoptysis, who may experience longer nonrecurrence periods after BAE. Tuberculosis sequelae and presence of aberrant bronchial artery or non-bronchial systemic collaterals were significant factors related to recurrent bleeding. The use of NBCA-based embolic materials significantly reduced the recurrent bleeding rate.

## Data Availability

The datasets during and/or analysed during the current study available from the corresponding author on reasonable request.

## Abbreviations

BAE: Bronchial artery embolization
CT: Computed tomography
PVA: Polyvinyl-alcohol
NBCA: N-butyl-2-cyanoacrylate
